# The burden of low back pain, rheumatoid arthritis, osteoarthritis, and gout and their respective attributable risk factors in Brazil: results of the GBD 2017 study

**DOI:** 10.1590/0037-8682-0285-2021

**Published:** 2022-01-28

**Authors:** Juliana Wolf, Elisabeth Barboza França, Ada Ávila Assunção

**Affiliations:** 1 Universidade Federal de Minas Gerais, Faculdade de Medicina, Programa de Pós-Graduação em Saúde Pública, Belo Horizonte, MG, Brasil.; 2 Universidade Federal de Minas Gerais, Núcleo de Estudos Saúde e Trabalho, Belo Horizonte, MG, Brasil.

**Keywords:** Low back pain, Rheumatoid arthritis, Osteoarthritis, Gout, Risk factors, Global Burden of Disease

## Abstract

**INTRODUCTION:**

Musculoskeletal (MSK) disorders are a major cause of disability worldwide. Different modifiable risk factors are associated to these disorders. The objective of this study was to analyze the burden of low back pain (LBP), rheumatoid arthritis (RA), osteoarthritis (OA), and gout, attributable to risk factors, in 2017.

**METHODS:**

The burden of LBP, RA, OA, and gout, and attributable risk factors were analyzed using data extracted from the Global Burden of Disease (GBD) Brasil-2017 study. Descriptive analysis was conducted to compare disability-adjusted life years (DALY) rates between sexes and age groups (15-49 and 50-69 years), in 2017.

**RESULTS:**

The highest rates of DALY due to LBP were attributed to occupational ergonomic factors in the 15-49-year group, regardless of sex and males aged 50-69 years, whereas smoking was the major contributor in the 50-69-year female group. RA-related DALY rates were attributed to smoking and were higher among women aged 50-69 years. High body mass index (BMI) was the most relevant risk factor for the burden of OA, with higher rates detected in the 50-69-year group, and it was the most significant risk factor for DALY rate attributed to gout, regardless of sex or age group.

**CONCLUSIONS:**

Occupational surveillance measures are indicated to prevent LBP. Actions to decrease smoking and overweight, and the surveillance of weight gain are warranted to decrease the burden of LBP, RA and OA, and gout, respectively. These actions will be more effective if age and sex differentials are considered in planning.

## INTRODUCTION

Musculoskeletal (MSK) disorders occur as a result of inflammatory or degenerative processes affecting musculoskeletal tissues. Different regions of the human body may be affected, such as the cervical and lumbar regions, and the upper and lower limbs^1^. Pain causes muscle stiffness and reduced mobility, leading to dependence, disability, and deformity^2^. Social interaction impairment, low levels of well-being and compromised work-related functions are dimensions of the impact of this disease^3^ ^,^ ^4^.

These disorders were the primary cause of duty-related disability retirement in 2017 (15,522 men and 14,972 women) in Brazilian urban areas, accounting for 17.4% of all retirements, and amounting to 45.5 million Brazilian *reals* in social security retirement benefits. The number of paid sick leaves granted to eligible urban and rural residents totaled up 329,347 and 37,435, respectively^5^. 

In the Global Burden of Disease (GBD) study, MSK disorders comprise all musculoskeletal system diseases, which are further divided into six subcategories: low back pain (LBP), rheumatoid arthritis (RA), osteoarthritis (OA), gout, neck pain (NP) and “others”, the latter including remaining MSK disorders subcategories^6^. The global incidence of MSK disorders increased between 1990 and 2017. However, the age-standardized incidence rate of these disorders has been declining^7^. 

Disability-adjusted life years (DALY) is a useful metric to determine the impact of MSK disorders and other morbidities in public health. DALY is a summary measure of the GBD study, which combines years of life lost (YLL) due to premature death and years lived with disability (YLD)^6^. 

MSK disorders, major cause of sickness absence worldwide, went from ranking 10^th^, in 2000, to 5^th^, in 2015, among 23 ICD-10 categories^8^. Data on the prevalence of risk factors have been provided in studies conducted with samples comprising specific professional categories^9^ ^-^ ^11^. However, studies investigating the Brazilian adult population are scarce^12^. 

In this study, we used data extracted from the GBD 2017 study to analyze the burden of LBP, RA, OA, and gout, attributable to risk factors according to sex and age group, in 2017.

## METHODS

### Study design and current analysis

This descriptive study was based on estimates of the GBD study, 2017. Data in that study were provided by the Institute for Health Metrics and Evaluation (IHME), from Washington University, Unites States (USA)^13^, and indicators were calculated and updated in the corresponding website^14^. 

The GBD 2017 study includes estimates for 359 diseases and injuries, 3484 sequelae and 84 risk factors, for 195 countries and territories. LBP, RA, OA, and gout are classified with the following codes according to the International Classification of Diseases (ICD-10): LBP (M54.3, M54.4 and M54.5), RA (M05, M06 and M08), OA (M16 and M17), gout (M10), NP (M54.2) and other MSK (L93, M00-M02, M08, M11-M13, M20-M25, M30-M35, M40-M43, M45-M46, M60 -M63, M65-M68, M70- M73, M75-M79, M80-M85, M86, M87-M90, M91-M94, M95-M99)^6^. Risk factors are grouped into three categories: behavioral, metabolic, and environmental/occupational comprising four levels each^15^. In the GBD study, the burden of MSK disorders and risk factors was estimated from Brazilian data obtained from several sources, including census, hospital records, national surveys, technical reports, and the scientific literature^16^. 

In this study, data were analyzed by sex and age group (15-49 and 50-69 years) for 2017. The estimates were presented in proportions, absolute number, and rates per 100,000 and their respective 95% uncertainty interval (UI)^17^. NP and “other MSK disorders” were not associated with risk factors examined in the GBD study. Therefore, these causes were not included in this analysis. All level 3 (occupational ergonomic factors, smoking, high body mass index (BMI), and impaired kidney function) risk factors as per the GBD study were accounted for. These risk factors meet the convincing or probable evidence of causation criteria established by the GBD 2017 study^15^. 

Occupational ergonomic factors were defined as the proportion of the working population exposed to low back pain-inducing work, based on population distributions across nine occupational categories, and the Theoretical Minimum Risk Exposure Level (TMREL) was assumed to be no exposure to that risk. Smoking was defined as current and former smoking of any tobacco product, and TMREL was zero smoking. High BMI was considered as BMI greater than 20 to 25 kg/m^2^ and the TMREL was determined based on the BMI level, which was associated with the lowest risk of all-cause mortality in prospective cohort studies. Impaired kidney function risk factor exposure was defined by urinary albumin to creatinine ratio (ACR) and estimated glomerular filtration rate (eGFR): albuminuria with preserved GFR, chronic kidney disease (CKD) stage 3, 4 and 5 . The TMREL for impaired kidney function was a diagnosis of albuminuria or CKD stages 3, 4, or 5^15^.

This study utilized only secondary databases which are publicly available, while respecting the ethical principles of Resolution no. 466/2012 of the Brazilian *Conselho Nacional de Saúde*. The GBD Brazil 2015 project was approved by the Research Ethics Committee of *Universidade Federal de Minas Gerais*, CAAE 62803316.7.0000.5149.

### GBD Study variables

For a given population, DALY are calculated as the sum of YLL due to premature death, and YLD, per age group, sex, location, and cause. The YLL is calculated as the estimated number of deaths at a particular age multiplied by the standard life expectancy at the age at which death occurs. The YLD is calculated as the prevalence of the disease multiplied by a weight factor attributed to each type of disability. Disability weights reflect the magnitude of health loss due to a disease, measured on a zero to one scale, where zero indicates full health and one is equivalent to death. The input data for all MSK disorders were obtained through scientific literature, surveys and vital registration. Adjustments were applied to extracted data to make it more consistent and suitable for modelling. Mortality was estimated using the Cause of Death Ensemble model (CODEm). This analytic tool generates a combined set of models for enhanced predictive performance regarding cause-specific mortality estimates^18^. Morbidity was estimated using the modelling system DisMod-MR 2.1^6^. Further methodological details are available^6^ ^,^ ^18^ ^,^ ^19^.

The burden attributable to risk factors expresses a hypothetic reduction in the current disease burden, which would be expected if an alternative or counterfactual distribution of previous exposure had been applied. Attributable burden estimates take into account four key components: burden quantification metric, levels of exposure to a risk factor, relative risk of a given exposure-related effect, and counterfactual risk factor exposure level. The TMREL concept was employed in the GBD study. Estimates of attributable burden, such as DALY for risk-outcome pairs, were calculated as the DALY multiplied by the population attributable fraction (PAF) for a risk-outcome pair for a given age, sex, location, and year. In short, PAF is the proportional risk reduction that would occur in a given year if previous exposure to a risk factor were reduced to the counterfactual level^15^. Detailed description of this methodology is available in published GBD studies^15^.

## RESULTS

In 2017, LBP was the major contributor to DALY due to MSK disorders among Brazilian men and women in both age groups considered, followed by other MSK disorders and NP. The proportional contribution of LBP was higher in the 15-49-year group (69.58% and 62.02%, men and women, respectively) compared to 50-69-year group, while NP, OA, RA, and gout prevailed in the 50-69-year group when compared to 15-49-year group (Table 1).

**TABLE 1:** Contribution of LBP, NP, RA, OA, gout and other MSK disorders to the total DALY caused by MSK disorders, by sex and age group. Brazil, 2017.

MSK disorders	Men						Women					
	15 to 49 years			50 to 69 years			15 to 49 years			50 to 69 years		
	Number	%	Rate*	Number	%	Rate*	Number	%	Rate*	Number	%	Rate*
LBP	808,481.18	69.6	1,432.53	396,333.67	57.5	2,207.16	797,216.82	62.0	1,376.49	440,896.14	53.5	2,174.62
NP	133,559.81	11.5	236.65	96,641.43	14.0	538.19	155,525.35	12.1	268.53	122,477.81	14.9	604.09
OA	19,968.94	1.7	35.38	55,470.57	8.0	308.91	21,042.11	1.6	36.33	62,842.04	7.6	309.95
RA	13,023.77	1.1	23.08	13,843.34	2.0	77.09	24,046.91	1.9	41.52	30,978.69	3.8	152.80
Gout	5,178.56	0.4	9.18	5,845.02	0.8	32.55	25,78.93	0.2	4.45	3,529.85	0.4	17.41
Other MSK disorders	181,727.32	15.6	322.00	121,273.83	17.6	675.37	284,994.68	22.2	492.08	162,662.82	19.8	802.30

*Rate per 100,000. **LBP:** low back pain; **NP:** neck pain; **RA:** rheumatoid arthritis; **OA:** osteoarthritis; **MSK:** Musculoskeletal; **DALY:** Disability-adjusted life years.

**FIGURE 1:**
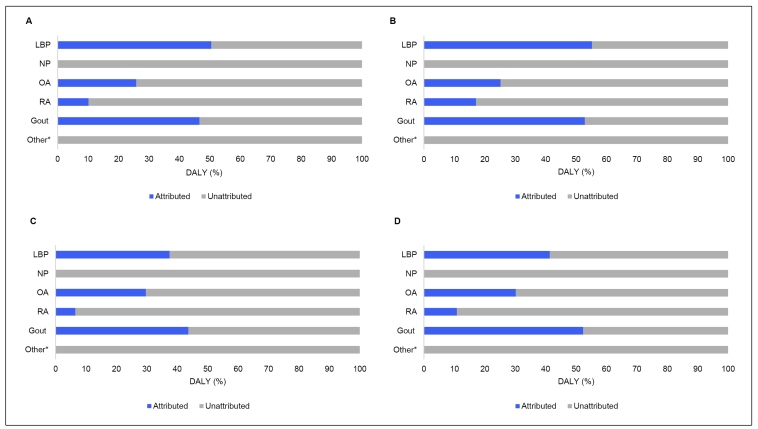
Proportion of low back pain (LBP), neck pain (NP), osteoarthritis (OA), rheumatoid arthritis (RA), gout and others MSK disorders attributable to risk factors by sex and age group. Brazil, 2017. **(A)** Men 15 to 49 years. **(B)** Men 50 to 69 years. **(C)** Women 15 to 49 years. **(D)** Women 50 to 69 years. *Other: Other MSK disorders.

The proportion of DALY that could be attributed to the risk factors currently assessed in GBD 2017, ranged from 52.89% for gout to 0% for NP and other MSK disorders (Figure 1). No association between risk factors and NP and other MSK disorders was identified. 

**FIGURE 2:**
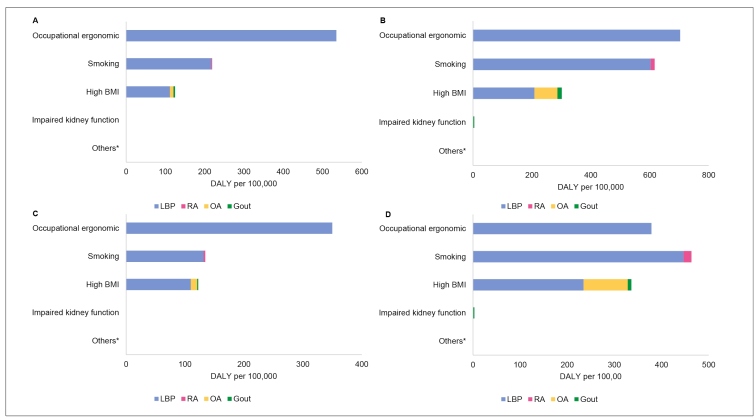
DALY rate caused by low back pain (LBP), osteoarthritis (OA), rheumatoid arthritis (RA), and gout attributable to level 3 risk factors, by sex and age group, per 100,000. Brazil, 2017. **(A)** Men 15 to 49 years. **(B)** Men 50 to 69 years. **(C)** Women 15 to 49 years. **(D)** Women 50 to 69 years. *Others: Unsafe water source; Unsafe sanitation; No handwashing with soap; Particulate matter pollution; Ambient ozone pollution; Residential radon; Lead exposure; Occupational carcinogens; Occupational asthmagens; Occupational particulate matter, gases, and fumes; Occupational noise; Occupational injuries; Suboptimal breastfeeding; Childhood undernutrition; Iron deficiency; Vitamin A deficiency; Zinc deficiency; Second-hand smoke; Alcohol use; Drug Use; Diet low in fruits; Diet low in vegetables; Diet low in whole grains; Diet low in nuts and seeds; Diet low in milk; Diet high in red meat; Diet high in processed meat; Diet high in sugar-sweetened beverages; Diet low in fiber; Diet suboptimal in calcium; Diet low in seafood omega-3 fatty acids; Diet low in polyunsaturated fatty acids; Diet high in trans fatty acids; Diet high in sodium; Intimate partner violence; Childhood sexual abuse; Bullying victimization; Unsafe sex; Low physical activity; High fasting plasma glucose; High LDL cholesterol; High systolic blood pressure; Low bone mineral density.

Occupational ergonomic risk was the risk factor that most contributed to the LBP burden among men (535.33 per 100,000) and women (349.88 per 100,000) aged 15-49 years. For women aged 50-69 years, smoking (446.73 per 100,000) was the main risk factor for LBP (Figure 2 **,** Table 2). 

**TABLE 2:** DALY rate and their respective 95% uncertainty interval (UI) caused by LBP, RA, OA, and gout attributable to level 3 risk factors, by sex and age group, per 100,000. Brazil, 2017.

Cause	Risk factors	Men				Women			
		15 to 49 years		50 to 69 years		15 to 49 years		50 to 69 years	
		Rate	UI 95%	Rate	UI 95%	Rate	UI 95%	Rate	UI 95%
LBP	Occupational ergonomic factors	535.33	367.77-755.14	703.15	457.21-1015.77	349.88	239.10-497.92	378.63	247.72-551.05
	Smoking	216.69	133.88-320.63	603.37	373.38-900.21	131.50	81.3-194.34	446.73	278.69-669.12
	High BMI	111.46	58.48-185.95	208.91	110.94-369.07	109.75	62.2-174.55	234.65	128.13-395.95
RA	Smoking	2.34	0.61-4.63	13.19	3.80-25.60	2.70	0.60-5.54	16.54	3.97-32.66
OA	High BMI	9.15	3.35-19.62	77.81	29.44-174.13	10.79	4.19-23.61	93.76	36.08-205.10
Gout	High BMI	4.10	1.94-7.40	14.77	7.12-25.46	1.83	0.97-2.98	7.49	3.95-12.04
	Impaired kidney function	0.31	0.19-0.48	4.45	2.83-6.38	0.20	0.12-0.31	2.86	1.81-4.12

**DALY:** disability-adjusted life years; LBP: low back pain; **RA:** rheumatoid arthritis; **OA:** osteoarthritis; **UI:** Uncertainty Interval.

The overall burden of RA was attributed to smoking. DALY rates were higher in the 50-69-year group among women (16.54 per 100,000). High BMI was the primary risk factor for the burden of OA. The rate of OA-related DALY attributed to high BMI was higher among women in both age groups, particularly 50-69 years (93.76 per 100,000) (Figure 2 **,** Table 2).

High BMI was also a major contributor to the gout burden attributable to risk factors in men and women, in both age groups. The rate of DALY due to gout attributable to high BMI was higher among men aged 50-69 years (14.77 per 100,000) (Figure 2 **,** Table 2).

## DISCUSSION

GBD 2017 showed LBP as the main cause of DALY among all MSK disorders, and it is mainly attributed to ergonomic risk factors. DALY related to RA, OA, and gout were mainly attributed to behavioral risk factors, including smoking for the RA and BMI for the other two. Regarding NP, it was not possible to describe the burden attributable to risk factors, because it was not associated with the risk factors assessed by the GBD study. The multifactorial nature of MSK disorders has been extensively documented^1^ ^,^ ^7^. Ergonomic risks and individual characteristics and lifestyles affect musculoskeletal function^20^. The proportion of DALY attributable to occupational ergonomic risk factors in this study was not surprising^21^ ^-^ ^24^. Statistical analyses adjusted for age, sex, level of education and duration of employment emphasized the impact of moderate to intense force application and repetitive work-related gestures on inflammatory and degenerative musculoskeletal processes^25^. Mechanical loading of soft tissues in response to these types of operations will be enhanced if workplace design, furniture and equipment selection do not fit the characteristics of individuals working in the setting^24^ ^,^ ^26^. Such ergonomic problems explain awkward postures adopted by operators while manipulating work-related items^22^, or the excessive force required to operate machinery and equipment, for instance^27^. The increased BMI reflects unbalanced diets and smoking, both of which are common behaviors in the contemporary *modus vivendi* ^7^. 

The evidence on environmental/occupational risk factors is relevant considering the magnitude of prevalence of LBP. It is known that LBP is experienced by approximately 50 to 80% of individuals at some point in life^28^, and it is the current leading cause of disability in countries where vital statistics are available, regardless of *per capita* income. YLD due to LBP increased 54% worldwide, from 1990 to 2015. These trends are partly explained by demographic changes, given the likelihood of suffering from LBP increases with age^2^ ^,^ ^29^. 

Smoking ranked first among risk factors for LBP in the 50-69-year women group. Chemicals in tobacco products cause vasoconstriction. In turn, decreased blood perfusion leads to intervertebral disc tissue malnutrition and development of degenerative intervertebral disk lesions responsible for low back symptoms. Tobacco chemicals are also a risk factor for osteoporosis and related LBP^30^. In the literature, gender-related disparities may be explained by exposure differences. In industries involving tasks associated with intense physical force use in awkward postures, the exposed population comprises primarily males^24^. Different from men of similar age, women aged 50-69 years are exposed to the effects of decreased estrogen levels on musculoskeletal tissues. There is evidence of severe intervertebral disk degeneration in post-menopausal women^31^ ^,^ ^32^. 

The association between high BMI and LBP can be explained by different mechanisms. Spinal overload in response to body weight gain puts localized pressure on tissues of this region. Obesity is associated with increased cytokine production and resulting activation of inflammatory processes. Obesity-related chronic systemic inflammation may be a cause of LBP^33^. 

RA is a systemic autoimmune condition with rising prevalence and age-standardized incidence rates^34^. Genetic and hormone factors, stress, obesity, infections and other factors have all been incriminated. However, smoking is widely recognized as the primary risk factor for this morbidity^35^, as shown in this study. Chemicals in cigarettes act as inflammatory mediators and play a significant role in development of aggressive joint damage. Although not gender-related, this effect is thought to be more pronounced in men^36^. Age and gender differentials were not observed in the RA burden. Regarding the occupational risks, they were not analyzed in GBD as already mentioned. 

OA manifests through a variety of clinical presentations and affects primarily women aged 50-69 years. The contribution of genetic factors, dietary habits and obesity to symptom development is widely recognized^37^. Despite the multifactorial nature of OA, obesity is thought to be a key factor in the development and progression of hip, knee, ankle, foot, and shoulder osteoarthritic lesions. Osteoarticular overload produced by excess body weight increases the risk of OA and bone fractures. Aside from the mechanical factor, inflammatory mediators produced by fat tissues are harmful to joint cartilages^38^. 

The burden of gout was attributed to high BMI. Similar findings have been reported in the GBD study comparing the global burden of this morbidity across different countries between 1990 and 2017^39^. High BMI was a contributing factor to rising gout rates among men and women. Potential relations between excessive dietary intake of red meats, seafood, and sweet foods and beverages, in countries with higher gout prevalence have been suggested. These foods are associated with higher levels of urate, typical of gout. Reduced dyslipidemia and serum urate levels in individuals who lost weight in response to changes in proportions of dietary macronutrients support this hypothesis^39^. 

MSK disorders affect a fifth of the Brazilian adult population. They are more prevalent in the population of the South region, among women, increased with advancing age and individuals with a low education level. Besides the risk factors addressed in this study, gender, age, education, insufficient physical activity, and the presence of morbidities can be associated with MSK disorders^40^. Age is one of the most common risk factors. Longer life expectancy and population growth may be related to an increase in the burden of MSK disorders^41^.

The prevalence of MSK disorders is expected to increase significantly over the next decades. This will likely translate into higher burden of disease^41^, especially in low- and middle-income countries^7^. MSK disorders share etiologic and aggravation risk factors with other chronic noncommunicable diseases (NCD). Ideal body weight, practice of regular physical activity, balanced diet, smoking cessation, and other healthy behaviors, need to be emphasized in programs aimed to tackle chronic NCD in Brazil.

However, recent results indicate the need to improve interventions in this area, because, despite the decrease in the prevalence of smoking since 1990^42^, 10% of Brazilian adults are still smokers^43^. Regarding overweight, the prevalence in the Brazilian adult population is higher among men (56.5% of men and 49.1% of women). The obesity prevalence is continuing to increase both in the Brazilian state capitals and the Federal District, indicating the need for revision of policies^44^.

Surprisingly, MSK disorders are not regarded as a priority and related environmental/occupational risk factors are not included in the national chronic NCD-tackling agenda^45^. Prevention approach to reduce MSK disorders should include healthy behaviors and ergonomically suitable work environment^46^. Also, timely access to the health system to identify and treat early and good-quality information on what can be done to prevent or effectively manage MSK disorders is needed^47^.

This study has limitations, which should be considered. First, the proportion of DALY not attributable to the risk factors assessed by the GBD Study is high. NP and other MSK disorders were not included in this analysis, because they were not associated with risk factors examined in the GBD study. The GBD results do not allow for comparison of all risk factors related to DALY for each type of MSK addressed. As noted for RA, OA, and gout, only behavioral risk factors are included. The literature associates exposure to ergonomic risk factors and RA, but the GBD data do not allow for further elaboration of these hypotheses^48^ ^,^ ^49^. This limit has consequences for interpretations of the results obtained in the present study. GBD data do not allow exploring the burden of neck pain, for example, attributable to occupational risks as shown in a study^50^. Furthermore, the entire burden of OA is not assessed, because only hip and knee OA is included in GBD Study. LPB estimates are based on self-reported data, which can result on bias. Another limitation is that the results depend on the quality and quantity of the input data for the models. Moreover, the 95% uncertainty interval of the estimates is large in several cases, and results should be taken with caution. Despite these limitations, this study advanced in analyzing the LBP, RA, OA, and gout attributable to risks factors for Brazil. To our knowledge, the results on the LBP burden attributable to ergonomic risks is pioneering in Brazil. The prevalence of these diseases in Brazil has been widely documented^9^ ^-^ ^12^ ^,^ ^51^. However, this is the first national study to compare LBP, RA, OA, and gout burden attributable to risk factors. This study revealed occupational ergonomic factors as an important risk factor to LBP. 

The findings of this study emphasized the need for better health and work sector policies, and suggested MSK disorders and occupational risk factors should be included in the agenda to address chronic NCD. Occupational surveillance measures are indicated in order to prevent LBP. Actions to decrease smoking and surveillance weight gain are warranted to decrease the burden of LBP, RA and OA, and gout, respectively. These actions will be more effective if age and sex differentials are considered in the planning. Determination of the burden of LBP, RA, OA, and gout, per Brazilian region and a continuing investigation of the burden attributable to environmental/occupational risks for NP, OA, RA, and gout are warranted. Furthermore, this study suggests the need for different gender approaches regarding the formulation of professional policies and actions aimed to prevent MSK disorders and respective subcategories.
